# To the Editors of the Medical and Physical Journal

**Published:** 1810-09

**Authors:** W. D. Rolfe

**Affiliations:** St. Augustine's Place, Bristol


### *To the Editors of the Medical and Physical Journal.*

Gentlemen,

A Correspondent having stated in your Journal for April, his Hints on Superfœtation, I beg leave to state the following case, that happened in my practice, for his observation.

My patient was a young woman, but had had one child before; she was very correct in her reckoning, and went to her full time. As soon as I came to her I found her labour going on regularly; the membranes soon ruptured, and in the course of a few pains, a long shrivelled fœtus was expelled, which I then concealed until I had an opportunity of examining it; the remainder of the labour went on very regularly, and was soon completed, by the birth of a fine large living child; the other appeared to me large enough for a six months fœtus, but was scarcely any thing but skin and bone, and of a dark colour, though without fœtor or any signs of putrefaction; and what appeared to me most singular, was, there was no appearance of a funis attached to it, nor any placenta that appeared to belong to it.

As the doctrine of conception has occasionally been discussed, allow me to insert the following case.

I was called to a Lady at the Hot Wells, who, I was informed by the practitioner first in attendance, had been in labour forty-eight hours, and for the last twelve hours the child's head had been pushing against the hymen, which was yet unbroken; after a few hours more patient waiting it gave way, but I could not complete the labour without the use of the forceps at last; and afterwards I questioned the husband respecting the hymen, who described to me very accurately, that it was the same as when he first married, and that he never had been able to enter the vagina completely.

I am, &c.

W. D. ROLFE.

*St. Augustine's Place, Bristol,*

*June* 1, 1810.

